# *In silico *structural analysis of quorum sensing genes in*Vibrio fischeri*

**Published:** 2015-09

**Authors:** Mohammed Zaghlool, Saeed Al-Khayyat

**Affiliations:** Biology Department, College of Education for Pure Sciences, University of Mosul, Mosul city, Iraq

**Keywords:** Quorum sensing, *Vibrio fischeri*, Motifs

## Abstract

Quorum sensing controls the luminescence of *Vibrio fischeri *through the transcriptional activator LuxR and the specific autoinducer signal produced by luxI. Amino acid sequences of these two genes were analyzed using bioinformatics tools. LuxI consists of 193 amino acids and appears to contain five α-helices and six ß-sheets when analyzed by SSpro8. LuxI belongs to the autoinducer synthetase family and contains an acetyltransferase domain extending from residues 24 to 110 as MOTIF predicted. LuxR, on the other hand, contains 250 amino acids and has ten α-helices and four ß-sheets. MOTIF predicted LuxR to possess functional motifs; the inducer binding site extending from amino acid residues 23 to 147 and the LuxR activator site extending between amino acids 182 and 236. The InterProScan5 server identified a winged helix- turn-helix DNA binding motif.

## INTRODUCTION

Environmental changes lead to morphological and physiological responses in bacteria through signal transduction pathways that send signals for transcription, causing gene expression to adapt the organism to the new environment. The signals originate from diffusible molecules synthesized by the cells themselves and serve to provide means for cell-to-cell communication and function as a mechanism for coordinating gene expression in response to cell population density in a phenomenon known as quorum sensing [1].

Signal molecules regulating quorum sensing are synthesized from cellular precursors by a synthase protein I and interact with a transcriptional activating R protein to provoke the expression of different target genes [2]. These signal molecules only act after bacterial population reaches a certain level so that the concentration of the signaling molecules reaches a threshold value [3].

Quorum sensing was first found to control the luminescence of *Vibrio fischeri*, a bacterium that forms symbiosis with certain marine animals to produce light [4, 5]. *V. fischeri *has been shown to regulate the expression of the *lux *operon through the transcriptional activator LuxR and the specific autoinducer produced by *luxI *[6]. This system exists in both Gram-negative and Gram-positive bacteria to control functions such as bioluminescence, virulence, biofilm formation and antibiotic production [7-9].

**Figure 1:**
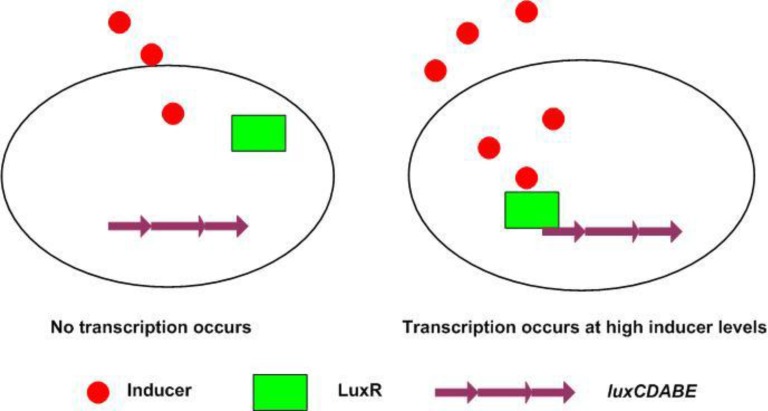
Quorum sensing involves two regulatory components: the transcriptional activator protein (R protein) and the inducer produced by the autoinducer synthase. Accumulation of the inducer takes place in a cell-density-dependent manner until a threshold level is achieved. At this time the inducer binds to and activates the R protein, which in turn causes gene expression (15).

In *V. fischeri*, quorum sensing is regulated by two proteins; LuxI and LuxR (Fig. 1) [10]. The LuxI protein is an autoinducer synthase responsible for the production of the autoinducer signal molecule, an acylated-homoserine lactone (AHL) which diffuses through the cell membrane [11]. The second protein, LuxR, is a regulatory protein which binds to the autoinducer and DNA [12]. Engebrecht and Silverman discovered both regulatory components (*luxI *and *luxR*) and the luciferase structural genes (*luxCDABE*) [10, 13]. In low concentrations, *V. fischeri *produces almost no light; however, when the cell density increases, the autoinducer accumulates so that a critical concentration of the inducer is reached, activating the expression of the *lux-ICDABE *operon. An exponential increase in autoinducer production occurs from the increase of *luxI *transcriptions, and due to the fact that the luciferase structural genes *luxCDABE *are located downstream to the *luxI*, an exponential increase in light production takes place [14]. The aim of the present study is to characterize the components of this system structurally and functionally.

## MATERIALS AND METHODS

Amino acid sequences, LuxI (Accesssion No. sp|P35328) and LuxR (Accession No. sp|P35327), were obtained from the uniprot database (available at http://www.uniprot.org) and formatted as FASTA files to be analyzed using the following online programs:

1- Compute pI/Mw, a tool which computes theoretical isoelectric points (pI) and molecular weights (Mw), available at http://web.expasy.org/compute_pi,

2- SSpro8 of SCRATCH, a program for predicting secondary and disordered regions, available at http://scratch.proteomics.ics.uci.edu/ [16]. The output of this program, according to Kabsch and Sander [17] is H: alpha-helix, G: 3-10-helix, E: extended strand, T: turn, S: bend, C: the rest,

3- PHYRE2, or Protein Homology/analog Y Recognition Engine, which determines protein tertiary structures, [18] available at http://www.sbg. bio.ic.ac.uk/phyre 2/html/ page.cgi?id= index,

4- MOTIF, a program to identify motifs from GenomeNet, Japan, using Pfam and Prosite databases, available at http:// www.genome. jp/tools/motif. This program depends on Pfam, a database of protein domain alignments derived from the protein sequence secondary database of the Swiss Institute of Bioinformatics (SWISS-Prot), and translates nucleic acid secondary databases stored in the European Molecular Biology Laboratory Database (TrEMBL) [19].

The *e*-value provides information about the likelihood of a given sequence match obtained by chance. This value is calculated by the program to indicate the probability of the motif in the sequenc significantly. If the *e*-value is small, the match is significant because it is less likely to be a result of random chance. If *e < *1 × 10−50, the database match is most likely to be a result of a homologous relationships. If *e *is between 0.01 and 1*×*10−50, the match can be considered as homology. If *e *is between 0.01 and 10, the match is considered insignificant, but may point to a tentative remote homology relationship [20].

5-InterProScan5, a program from the European Bioinformatics Institute, United Kingdom, available at http://www.ebi.ac.uk/interpro. InterProScan 5 uses several databases such as PROSITE, Pfam, PRINTS, ProDom and SMART to identify signature protein motifs [21].

## RESULTS AND DISCUSSION

**Figure 2:**
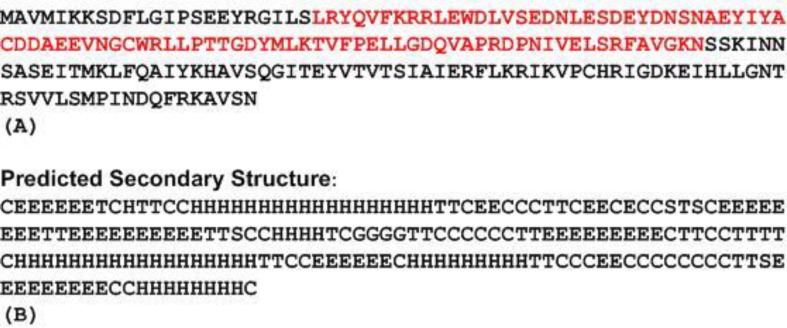
(A) The amino acid sequence of LuxI in *V.* *fischeri* ES114 showing acetyltransferase domain in red (B) predicted secondary structure by SSpro8 where H: alpha-helix, G: 3-10-helix, E: extended strand, T: turn, S: bend, C: the rest

**Figure 3:**
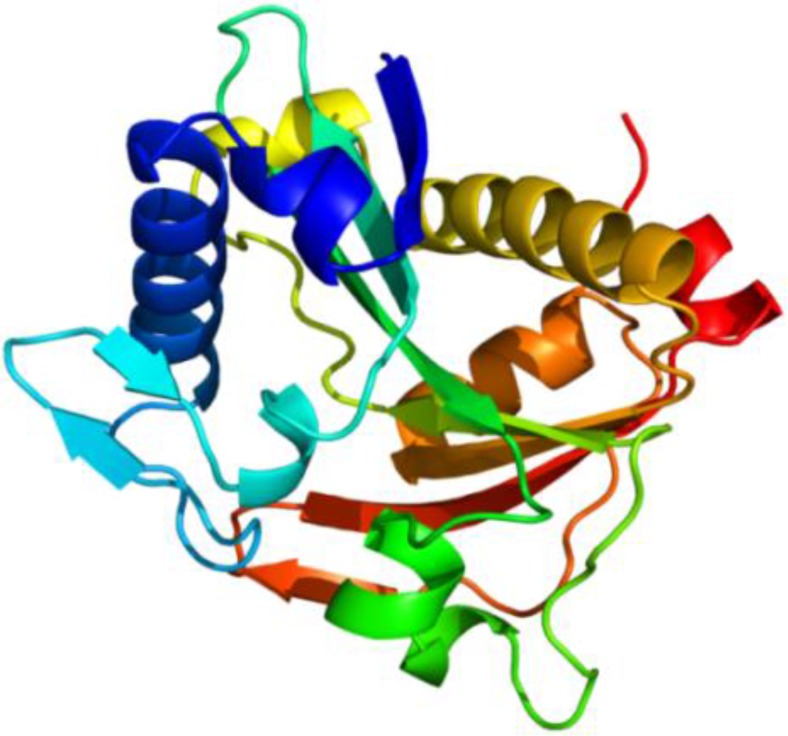
LuxI tertiary structure is composed of five α-helices and six β-strands connected by loops as predicted by Phyr2

LuxI consists of 193 amino acids and has Mw of 22014.15 Dalton and pI of 5.70 as estimated by the Compute pI/Mw software online. Using the SSpro8 program that predicts secondary structure (Fig. 2B), it appears that the molecule consists of five distinct α helices: α1 15→32, α2 79→82, α3 120→137, α4 149→157 and α5 187→195 and six ß sheets: ß1 2→7, ß2 54→61, ß3 64→73, ß4 102→109, ß5 142→147, ß6 176→184. Phyre2 was used to predict the tertiary structure (Fig. 3).

To predict functional motifs and domains in LuxI, MOTIF software was used. Results show that LuxI had an autoinducer synthetase family signature domain (e- value=3.1×10-34) and contained an acetyltransferase domain extending from residues 24 to 110 (e-value = 9.9×10-9) (Fig. 2A).

Members of the LuxI family of proteins are synthases that catalyze the production of acylated homoserine lactones (acyl-HSLs). The acyl portion of the acyl-HSL is derived from fatty-acid precursors conjugated to the Acyl carrier protein (acyl-ACP), and the HSL moiety is derived from *S*-adenosylmethionine (SAM) [22, 23]. The LuxI enzyme promotes the formation of an amide bond joining the acyl side chain from the acyl-ACP to SAM. Lactonization of the ligated intermediate together with the subsequent release of methylthioadenosine (MTA) results in the formation of acyl-HSLs [22].

Many different LuxI type proteins which were 190–230 amino acids long and shared 30–35% similarity were identified in Proteobacteria. Ten residues were conserved within most LuxI proteins in the amino terminal 110 amino acids, but no correlation was found between the synthesized acyl-HSL and the level of sequence similarity among the proteins. Nevertheless, it has been previously noted that many of the LuxI proteins that direct the synthesis of 3-oxoacyl-HSLs have a conserved threonine residue at the 143 position [24].

Recent structural and mutational analyses of the acyl-HSL synthase from *Pantoea stewartii *indicates that this threonine residue might be involved in stabilizing interactions with fatty-acyl biosynthetic precursors carrying a carbonyl group at the third position in the acyl chain [25]. Crystal structures have shown that acyl-HSL synthases have structural similarities with *N-*acetyltransferases of eukaryotes [24].

**Figure 4:**
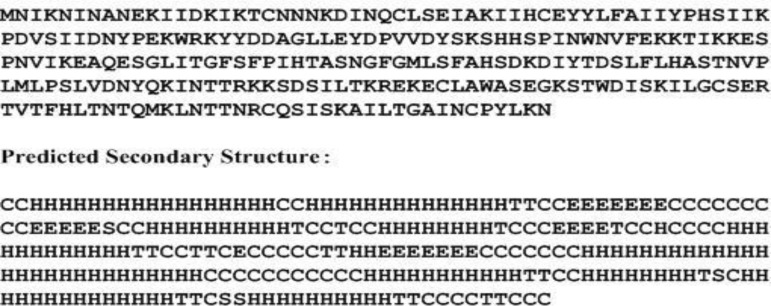
Amino acid sequence of LuxR and secondary structure of LuxR predicted by SSpro8. H:alpha-helix, E: extended strand, T: turn, S: bend, C: the rest.

**Figure 5:**
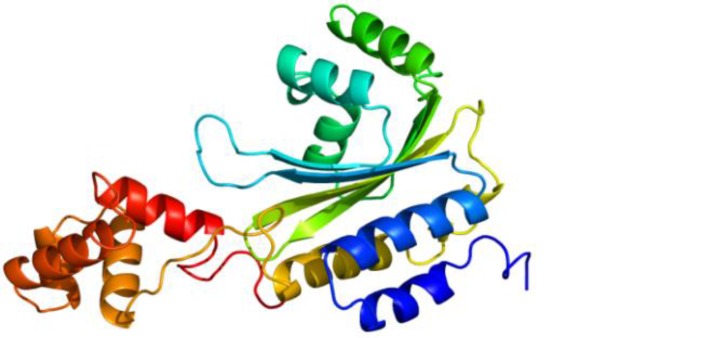
LuxR tertiary structure is composed of ten α-helices and four β-strands connected by loops as predicted by Phyr2.

LuxR, on the other hand, contains 250 amino acids (Fig. 4A), and has an estimated Mw of 28519.63 Dalton and pI of 8.54. The molecule has ten α helices: α1 3→19, α2 22→35, α3 64→73, α4 80→87, α5 104→115, α6 147→173, α7 185→195, α8 200→207, α9 211→224 and α10 230→239 and four ß sheets: ß1 40→46, ß2 56→60, ß3 92→95 and ß4 133→139 [Fig. 4B]. Phyr2 demonstrated the tertiary structure prediction of the molecule (Fig. 5).

MOTIF was also used to analyze LuxR (Table 1). The results indicate that the inducer binding site extended from amino acid residues 23 to 147, while the LuxR activator site extended between amino acids 182 and 236. The homeodomain fold is a protein structural domain that binds DNA and is found in transcription factors. The fold consists of a 60-amino acid helix-turn-helix structure on which three alpha helices are connected by short loop regions. The two N-terminal helices are antiparallel, and a longer C-terminal helix is perpendicular to them, interacting directly with the DNA [26, 27].

**Table 1:** Functional motifs in LuxR predicted by MOTIF program

**Motif**	**Position/(e-value)** *	**Recognition sequence**
Autoinducer binding domain	23...147/ (1.3×10-26)	KDINQCLSEIAKIIHCEYYLFAIIYPHSIIK PDVSIIDNYPEKWRKYYDDAGLLEYDP VVDYSKSHHSPINWNVFEKKTIKKESPN VIKEAQESGLITGFSFPIHTASNGFGMLSFAHSDK DIYT
Bacterial regulatory proteins, luxR family	182...236/ (1.2×10-20)	ILTKREKECLAWASEGKSTWDISKIL GCSERTVTFHLTNTQMKLNTTNRCQSISK
Homeodomain-like domain	191...215/ (0.002)	LAWASEGKSTWDISKILGCSERTVT
Sigma-70, region 4	183...219/ (0.0051)	LTKREKECLAWASEGKSTWDISKILGCSERT VTFHLT
Helix-turn-helix domain	190...214/ (0.48)	CLAWASEGKSTWDISKILGCSERTV
HTH DNA binding domain	201...225/ (0.41)	WDISKILGCSERTVTFHLTNTQMKL

* *e*-value represent the probability that a sequence could arise randomly by chance, values below 0.01 could be of random appearance.

DNA-binding proteins play a crucial role in the biology of the cell, being responsible for the transfer of biological information from genes to proteins [28, 29]. A large number of DNA-binding proteins are deposited in the Protein Data Bank (PDB) [30] and the Nucleic Acid Database [31]. HTH is a short motif consisting of a first alpha- helix, a connecting turn and a second recognition helix which interacts with the DNA. The two alpha-helices extend from the domain surface and make a convex unit to fit into the major groove of DNA [23, 33, 34].

Protein families with DNA-binding HTHs are greatly diverged, showing immense variation in amino acid sequences, sequence portions of DNA-binding domains and structural elements outside the DNA-binding motif [35]. It can be concluded from the analysis performed by MOTIF that LuxR possesses a regulatory domain extending along the C-terminal region, and an HTH structure which binds DNA and contains a sequence similarity to region 4 of the sigma factor belonging to RNA polymerase.

LuxR is a member of a family of transcriptional activators defined by sequence similarities in a C-terminal helix-turn-helix containing region [36]. Previous studies indicated two regions of the LuxR protein necessary for activity; one extending between residues 79-127 involved in autoinducer binding, and the other, between residues 184-230, supposedly involved in DNA binding [37, 38].

Residues 184-230 form the helix-turn-helix, which is a highly conserved region characterizing the LuxR family [36]. The C-terminal region of the LuxR family members also shows significant sequence similarity to region 4 bacterial RNA polymerase σ factors [39, 40]. Region 4 is a helix-turn-helix-containing region considered to recognize the -35 sequences of promoters [41].

InterProScan 5 program was used to explore further the different motifs in LuxR shown by MOTIF. The results showed four motifs; (1) transcription regulator LuxR, C- terminal (2) transcription factor LuxR-like autoinducer-binding domain (3) winged helix-turn-helix DNA binding motif (4) signal transduction response regulator, C- terminal effector, (which is an unrelated motif since it is a fragment of the two- component signal transduction system). Accordingly, LuxR is a transcription regulator containing an autoinducer binding site and of winged helix-turn-helix configuration.

The winged helix proteins constitute a subfamily of helix-turn-helix proteins. A large number of such related proteins with diverse biological functions have been characterized by X-ray crystallography and solution NMR spectroscopy. Studies of winged helix proteins and their complexes with DNA have shown that the motif isextremely variable, exhibits two different modes of DNA binding, and can participate in protein–protein interactions [42].

**Figure 6:**
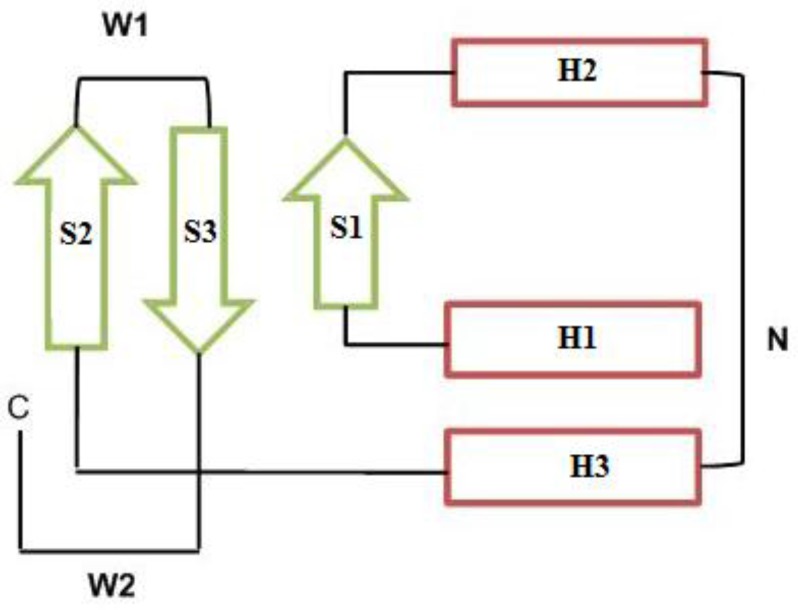
Winged helix-turn-helix topology consists of two wings: W1 and W2, three α-helices (H1, H2 and H3) and three ß-strands (S1, S2 and S3). The N-terminal half (N) is helical, whereas the C- terminal half (C) is composed of the twisted antiparallel ß-sheet and the two large wings (see text below) (41

The winged helix motif is a compact α/ß structure composed of two wings (W1 and W2), three α-helices (H1, H2 and H3) and three ß-strands (S1, S2 and S3), arranged in the order H1-S1-H2-H3-S2-W1-S3-W2 (Fig. 6). The N-terminal half of the motif is helical, whereas the C-terminal half is composed of two of the three strands forming the twisted antiparallel ß-sheet and the two large loops or wings, W1 and W2. Wing W1 connects strands S2 and S3, and wing W2 extends from strand S3 to the C terminal of the DNA binding domain. These loops flank helix H3 like the wings of a butterfly, inspiring the name “winged helix motif” [43].

The results of this study show that the quorum sensing system of *V. fischeri *is composed of two proteins; the LuxI, a synthease which produces the autoinducer and contains the Acyltranseferase motif, and the LuxR, which is a transcription regulator containing LuxR-like autoinducer-binding sites and possess a winged helix-turn-helix DNA binding domain.
